# Opposing roles of σ^B ^and σ^B^-controlled SpoVG in the global regulation of *esxA *in *Staphylococcus aureus*

**DOI:** 10.1186/1471-2180-12-17

**Published:** 2012-01-24

**Authors:** Bettina Schulthess, Dominik A Bloes, Brigitte Berger-Bächi

**Affiliations:** 1 Institute of Medical Microbiology, University of Zurich, Gloriastrasse 32, 8006 Zurich, Switzerland; 2 Cellular and Molecular Microbiology Division, Interfaculty Institute of Microbiology and Infection Medicine, University of Tübingen, Elfriede-Aulhorn-Strasse 6, 72076 Tübingen, Germany

## Abstract

**Background:**

The production of virulence factors in *Staphylococcus aureus *is tightly controlled by a complex web of interacting regulators. EsxA is one of the virulence factors that are excreted by the specialized, type VII-like Ess secretion system of *S. aureus*. The *esxA *gene is part of the σ^B^-dependent SpoVG subregulon. However, the mode of action of SpoVG and its impact on other global regulators acting on *esxA *transcription is as yet unknown.

**Results:**

We demonstrate that the transcription of *esxA *is controlled by a regulatory cascade involving downstream σ^B^-dependent regulatory elements, including the staphylococcal accessory regulator SarA, the ArlRS two-component system and SpoVG. The *esxA *gene, preceding the *ess *gene cluster, was shown to form a monocistronic transcript that is driven by a σ^A ^promoter, whereas a putative σ^B ^promoter identified upstream of the σ^A ^promoter was shown to be inactive. Transcription of *esxA *was strongly upregulated upon either *sarA *or *sigB *inactivation, but decreased in *agr*, *arlR *and *spoVG *single mutants, suggesting that *agr*, ArlR and SpoVG are able to increase *esxA *transcription and relieve the repressing effect of the σ^B^-controlled SarA on *esxA*.

**Conclusion:**

SpoVG is a σ^B^-dependent element that fine-tunes the expression of *esxA *by counteracting the σ^B^-induced repressing activity of the transcriptional regulator SarA and activates *esxA *transcription.

## Background

The production of virulence factors in *Staphylococcus aureus *is coordinated by a network of two-component systems, global regulators and transcription factors, allowing optimal adaptation of the pathogen to a changing environment and stress conditions encountered during the various stages of infection. A central regulatory element of virulence factor production in *S. aureus *is the accessory gene regulator *agr*, a two-component quorum sensor regulating gene expression in a growth-dependent manner. The main effector molecule of the *agr *operon is the regulatory RNAIII [1], which is responsible essentially for the upregulation of secreted proteins in the post-exponential phase. RNAIII transcription is enhanced by the staphylococcal accessory regulator SarA [2] and reduced by the alternative sigma factor σ^B ^in strain Newman [3,4]. SarA is a winged helix transcription factor influencing many virulence genes [5,6]. The transcription of *sarA *in turn is directly activated by the alternative sigma factor σ^B ^[3], which controls over 250 genes including virulence factors and secondary regulators in a direct or indirect way [7]. Indirectly σ^B^-controlled genes lack a σ^B ^consensus promoter sequence, and are thought to be controlled by secondary, σ^B^-dependent regulatory elements. The *yabJ-spoVG *operon, with SpoVG as effector molecule, is besides SarA one of the directly σ^B ^-dependent secondary regulators [8]. SpoVG contributes to methicillin and glycopeptide resistance, stimulates capsule synthesis, and was recently shown to regulate a small σ^B^-subregulon comprising mainly excreted virulence factors including the highly upregulated virulence factor EsxA [8-10].

Secretion of virulence factors is facilitated by several translocation systems in *S. aureus *[11], the major Sec pathway, the accessory Sec2 system [12], the twin-arginine translocation pathway [13], and the type VII-like specialized ESX secretion pathway (Ess) [14]. The Ess system comprises a cluster of at least nine genes: *esxAB, essABC, esaABC *and *esaD *[14,15] and secretes proteins with a size of approximately 100 amino acids containing a helical structure and a conserved Trp-Xaa-Gly (WXG) motif [16]. Three proteins were so far shown to be exported by the staphylococcal Ess system, two WXG100 family proteins, EsxA and EsxB, and the non-WXG100 substrate EsaC [14,17]. All three proteins act as pathogenicity factors in a murine model of staphylococcal blood-borne dissemination and abscess formation [14,17]. The actual role of EsxA, EsxB and EsaC remains unclear. Structural analysis of EsxA suggests a role as transport module or chaperone to assist export of proteins by the Ess secretion pathway rather than being an effector protein itself [18]. The *esxA *gene seems to be under complex control. Besides being upregulated by SpoVG [10], *esxA *was found to be upregulated by ArlR [19]. The two-component system ArlRS [19,20] itself is activated in an indirect way by σ^B ^in strain Newman [3,9], adding a further level of complexity in the regulation of *esxA*.

This study analyses the transcriptional control of *esxA *by σ^B ^and the σ^B^-dependent regulatory elements SarA, ArlR, RNAIII and SpoVG.

## Materials and methods

### Bacterial strains, plasmids and culture conditions

The bacterial strains and plasmids are listed in Table 1. Bacteria were grown on Luria Bertani (LB) agar (Becton Dickinson, Franklin Lakes, NJ, USA) or in LB broth with shaking (180 rpm) at 37°C in a flask to medium ratio 5:1. Where required, media were supplemented with 100 μg ml^-1 ^ampicillin, 20 μg ml^-1 ^chloramphenicol, 10 μg ml^-1 ^erythromycin, or 10 μg ml^-1 ^tetracycline.

**Table 1:** Strains and plasmids used in this study

Strain or plasmid	Relevant genotype; phenotype	Reference or source
***S. aureus***		

Newman	Clinical isolate, ATCC 25904, natural *saeS *constitutive mutant	[21,22]

BS304	Newman Δ*esxA*, markerless deletion	This study

SM148	Newman Δ(*yabJ-spoVG)::erm*(B); Em^r^	[8]

IK184	Newman Δ(*rsbUVW-sigB)::erm*(B); Em^r^	[23]

MS64	Newman *sigB1*(Am); Tc^r^	[24]

SM99	Newman Δ*arlR::cat*; Cm^r^	[9]

BS310	Newman Δ*agr::ermB*; Em^r^	This study

KS186	Newman Δ*agr::tet(M)*; Tc^r^	[25]

BS309	Newman Δ*sarA::ermB*; Em^r^	This study

LR15	Newman Δ*sarA::tet(L)*; Tc^r^	L. Reutimann

BB1002	Newman *mec*, MRSA derivative, Mc^r^	[26]

BS307	BB1002 Δ*esxA*, markerless deletion	This study

NM143	Newman GISA derivative, in vitro selected mutant; Te^r^	[27]

BS308	NM143 Δ*esxA*, markerless deletion	This study

***E. coli***		

DH5α	F^-^Φ80d/acZΔM15 *recA1*	Invitrogen

**Plasmids**		

pKOR1	*E. coli-S. aureus *shuttle vector for markerless deletions using the counter selection system	[28]

pAC7	Expression plasmid containing the P_BAD _promoter and the *araC *gene; Cm^r^	[29]

pAC7-*sigB*	pAC7 with a 0.75 kb fragment containing the gene *sigB *from *S. aureus *Col; Cm^r^	[30]

pBus1	*E. coli-S. aureus *shuttle plasmid with multicloning site from pBluescript II SK (Stratagene) and the rrnT14 terminator sequence from pLL2443; Tc^r^	[31]

p*yabJ*	pBus1 containing a *bacA *promoter-*yabJ *ORF fusion construct; Tc^r^	[10]

p*spoVG*	pBus1 containing a *bacA *promoter-*spoVG *ORF fusion construct; Tc^r^	[10]

p*yabJspoVG*	pBus1 containing a *bacA *promoter-*yabJ*-*spoVG *operon fusion construct; Tc^r^	[10]

pSP*-luc^+^*	Firefly luciferase casette vector; Ap^r^	Promega

p*esxAp-luc^+^*	pBus1 containing an *esxAp*-*luc*^+^ fusion fragment; Tc^r^	This study

p*esxAp*Δσ^A^*-luc^+^*	p*esxAp-luc^+ ^*with deletion of the σ^A ^promoter	This study

p*esxAp*Δσ^B^*-luc^+^*	p*esxAp-luc^+ ^*with deletion of the σ^B ^promoter	This study

pSB40N	Promoter probe plasmid; Ap^r^	[29]

p*asp23p*	pSB40N with a 0.6 kb fragment covering the *asp23 *promoter region fused to the reporter gene *lacZα*; Ap^r^	[30]

p*esxAp*	pSB40N with a 0.5 kb fragment covering the *esxA *promoter region fused to the reporter gene *lacZα*; Ap^r^	This study

p*yabJp*	pSB40N with a 0.4 kb fragment covering the *yabJ *promoter region fused to the reporter gene *lacZα*; Ap^r^	This study

pSTM07	pSB40N with a 0.37 kb fragment covering the *capA *promoter region fused to the reporter gene *lacZα*; Ap^r^	[9]

^*a *^Abbreviations are as follows: Ap^r^, ampicillin resistant; Cm^r^, chloramphenicol resistant; Em^r^, erythromycin resistant; Mc^r^, methicillin resistant; Tc^r^, tetracycline resistant; Te^r^, teicoplanin resistant.

### Molecular biological methods

General molecular biology techniques were performed according to standard protocols [32,33]. Sequencing was done using the Big Dye Terminator Cycle Sequencing Ready Reaction Kit and an ABI Prism 310 genetic analyzer (Applied Biosystems, Foster City, CA, USA). Sequences were analyzed with the Lasergene software package (DNASTAR, Inc., Madison, WI, USA).

### Construction of Δ*esxA *mutants

The markerless deletion of *esxA *(*nwmn_0219*) in strains Newman, BB1002 and NM143 was constructed using the counter selection system of pKOR1 as described by Bae et al. [28], using primer pairs oBS43/oBS44 and oBS45/oBS46 (Table 2) to amplify sequences framing *esxA*. Correct deletion of *esxA *in BS304, BS307 and BS308, respectively, was confirmed by sequencing and Southern blot analysis, and the absence of major rearrangements by pulsed-field gel electrophoresis [34].

**Table 2:** Oligonucleotide primers used in this study

Primer name	**Sequence (5'-3')**^***a***^	reference
***esxA *deletion**		

oBS43	GGGGACAAGTTTTGTACAAAAAAGCAGGCTacgtttatcaaagacatacc	This study

oBS44	gggggtaccaactagaaacctcctgaata	This study

oBS45	gggggtaccgcattctgaaattggcaaag	This study

oBS46	GGGGACCACTTTGTACAAGAAAGCTGGGTttatccatcgctgtattgtg	This study

**DIG probes**		

Nwmn0219-DIG-f	tccagaggaaatcagagcaaa	[10]

Nwmn0219-DIG-r	cttgttcttgaacggcatca	[10]

oSTM29 (*spoVG*f)	gcgtcgacttattgcaaatgtattacatcgc	[9]

oSTM43 (*spoVG*r)	gcggagctccactcgtttccattacattagatg	[8]

Sa*sarA*f	agggaggttttaaacatggc	[35]

Sa*sarA*f	ctcgactcaataatgattcg	[35]

RNAIII+	gtgatggaaaatagttgatgag	[3]

RNAIII-	gtgaatttgttcactgtgtcg	[3]

arlRSprobe+	tcgtatcacatacccaacgc	[36]

arlRSprobe-	gagtatgatggacaagacgg	[36]

SA*asp23*+	atgactgtagataacaataaagc	[37]

SA*asp23*-	ttgtaaaccttgtctttcttgg	[37]

**plasmid construction**		

Pnwmn0219F	tgcggatccgatcacgttgatttgcgtgt	This study

Pnwmn0219R-xho	tgcctcgagctagaaacctcctgaatattttaag	This study

yab-prom-bam-f	gcgggatcctgctaatattttaaatttacc	This study

yab-prom-xho-r	gcgctcgagtactaaaactccttttatgaaaac	This study

Pnwmn0219F-hind	tgcaagcttgatcacgttgatttgcgtgt	This study

Pnwmn0219R	tgcccatggctagaaacctcctgaatattttaag	This study

pSP-Luc XhoI	accggcctcgagatcgatgatatcgaa	This study

oBS49	tagttttttaagtatttttagtttttttta	This study

oBS51	attcaatatatttatttaaaaaaaactaaaaa	This study

oBS53	aggtaccttgagtaaggagcactttttcaa	This study

oBS54	aggtaccattcatttttgtaatataaatgtgtatac	This study

**primer extension**		This study

pe_esxA_1	BIOTIN-ccataactagaaacctcctg	This study

pe_esxA_2	BIOTIN-tgatttcctctggactcatc	This study

esxA_term-r	tgcggtaccatgcttatttcctttcagttg	This study

^*a *^Restriction sites are underlined. Capital letters show the att sites.

### Construction of BS309 and BS310

The Newman *sarA *mutant BS309 and the Newman *agr *mutant BS310 were constructed by transducing the *ermB*-tagged *sarA *mutation of NM520 [38], and the *ermB*-tagged *agr *mutation of NM521 [38] respectively, into Newman and selecting for erythromycin resistance. Correct inactivation of the genes was confirmed by sequencing and Southern blot analysis.

### Plasmid construction

For the construction of promoter-*lacZ *reporter fusions, DNA fragments covering the *yabJ *or *esxA *promoter of strain Newman were amplified using primer pairs yab-prom-bam-f/yab-prom-xho-r and Pnwmn0219F/Pnwmn0219R-xho (Table 2), respectively. The PCR products were digested with BamHI and XhoI and ligated into promoter probe plasmid pSB40N [29] upstream of the *lacZα *reporter gene to obtain p*yabJp *and p*esxAp*.

For the construction of p*esxAp-luc*^*+ *^, the *esxA *promoter region of strain Newman was amplified by PCR using primer pair Pnwmn0219F-hind/Pnwmn0219R (Table 1). The resulting PCR product was HindIII/NcoI-digested and cloned into pSP-*luc^+ ^*upstream of the luciferase reporter gene *luc^+ ^*. The *esxA *promoter-*luc^+ ^*fusion of the resulting plasmid was amplified using the primers Pnwnm0219F-hind/pSP-Luc XhoI, digested with HindIII and XhoI and cloned into the *E. coli*-*S. aureus *shuttle plasmid pBus1 to obtain plasmid p*esxA-luc^+ ^*.

Plasmids p*esxAp*Δσ^A^*-luc^+ ^*and p*esxAp*Δσ^B^*-luc^+ ^*were made by deleting the σ^A ^and σ^B ^promoter sequences, respectively, from p*esxAp-luc^+ ^*. The corresponding DNA fragments were amplified with primer pairs oBS49/oBS53 and oBS51/oBS54 (Table 2) from p*esxAp-luc^+ ^*and religated.

All plasmids constructs were confirmed by sequence analyses.

### Northern blot analysis

Overnight cultures were diluted 1:100 into LB, grown for 2 h, and then used to inoculate 100 ml of pre-warmed LB to an optical density of 600 nm [OD_600 nm_] of 0.05. Cell samples were taken at the time points indicated, centrifuged at 12,000 × g and 4°C for 2 min, the pellets were snap-frozen in liquid nitrogen. Total RNA was isolated according to Cheung et al. [39]. RNA samples (8 μg) were separated in a 1.5% agarose gel containing 20 mM guanidine thiocyanate in 1 × Tris-borate-EDTA buffer [40]. RNA transfer and detection were performed as previously described [41,42]. Digoxigenin (DIG) labelled probes were amplified using the PCR DIG Probe synthesis kit (Roche, Basel, Switzerland). The primer pairs used for amplification of the *esxA*, *spoVG*, *asp23*, *arlR*, *sarA *and RNAIII probes are listed in Table 2.

### Primer extension

RNA was extracted from LR15 cultures that were grown to OD_600 nm _2.0, as described by Cheung et al. [39]. Primer extension reactions were performed using 20 μg of total RNA and 3 pmol of the 5'-biotin-labelled primers pe_esxA_1 and pe_esxA_2 (Table 2) using Superscript II reverse transcriptase (Invitrogen, Carlsbad, CA, USA), according to the manufacturers instructions. Sequencing reactions were performed using the Thermo Sequenase Cycle Sequencing Kit (USB Corporation, Cleveland, OH, USA) and template DNA amplified with primers Pnmmn0219F and esxA_term-r from Newman genomic DNA. The Biotin Chromogenic Detection Kit (Fermentas, Burlington, Ontario, Canada) was used for biotin detection.

### Two-plasmid testing

Testing of the interaction of *S. aureus *promoters with *E. coli *RNA polymerase containing *S. aureus *σ^B ^was done essentially as described earlier [30]. The promoter-reporter plasmids p*asp23p *(*asp23 *promoter); p*yabJp *(*yabJ *promoter); p*esxap *(*esxA *promoter); and pSTM07 (*capA *promoter); or the empty plasmid pSB40N, were transformed into *E. coli *DH5α containing either pAC7-*sigB *or pAC7. The color production of the clones was analyzed on LBACX-ARA plates (LB agar containing 5 mg ml^-1 ^lactose; 100 μg ml^-1 ^ampicillin; 40 μg ml^-1 ^chloramphenicol; 20 μg ml^-1 ^X-Gal (5-bromo-4-chloro3-indolyl-D-galactopyranoside) and 2 μg ml^-1 ^arabinose) [29].

### Luciferase assay

Luciferase activity was measured as described earlier [3] using the luciferase assay substrate and a Turner Designs TD-20/20 luminometer (Promega).

### Protease activity

The proteolytic activity of *S. aureus *strains was determined on skim milk (Becton Dickinson, 75 g l^-1^) agar plates as clear zones surrounding colonies.

### Hemolytic activity

To compare the hemolytic activity, *S. aureus *strains were grown on sheep blood agar and the clear halos around the colonies were analyzed.

### Susceptibility testing

Plates containing an antibiotic gradient were prepared and inoculated by swabbing a 0.5 McFarland cell suspension in physiological NaCl solution along the gradient as described before [27]. Growth was read after 24 h and 48 h of incubation at 35°C. Teicoplanin and oxacillin minimal inhibitory concentrations (MICs) were determined using Etests according to the manufacturer's instructions (AB-Biodisk, Solna, Sweden).

## Results and discussion

### Transcriptional analysis of *esxA*

**Figure 1:**
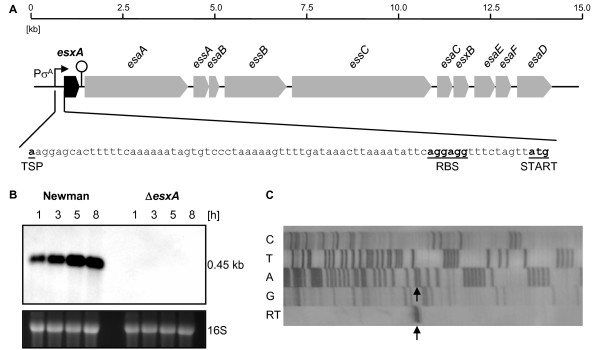
***esxA *in *S. aureus***. **A**. Schematic representation of the *ess *locus of *S. aureus *Newman (GenBank accession no. NC_009641). ORF notations correspond to those used by Anderson et al. [15]. The σ^A ^promoter, transcriptional start point (TSP) and ribosomal binding site (RBS) as well as the start codon of *esxA *are indicated. **B**. Northern blot of *esxA *of strain Newman and the isogenic Δ*esxA *mutant (BS304) during growth. The ethidium bromide-stained 16S rRNA pattern is shown as an indication of RNA loading. **C**. Primer extension analysis of *esxA*. Lanes C, T, A and G show the dideoxy-terminator sequencing ladder and lane RT the reverse transcription product obtained using primer pe_esxA_2. The TSP is marked by an arrow. The same TSP was identified using primer pe_esxA_1 (data not shown).

The 294 bp *esxA *gene (*nwmn_0219*, GenBank accession no. NC_009641), coding for a small secreted protein involved in staphylococcal virulence, is the first of at least nine genes of the *ess *gene cluster encoding the type VII-like ESX-1 secretion pathway (Ess) in *S. aureus *(Figure 1A) [14,15]. Although *esxA *seems to belong transcriptionally to the *ess *gene cluster [43], transcriptional profiling produced one single *esxA*-specific transcript with a size of about 0.45 kb appearing in early growth phase after 1 h and increasing slightly within time (Figure 1B). No *esxA*-specific signals were detected in the corresponding Δ*esxA *mutant BS304, confirming the *esxA *deletion. The deletion of *esxA *had no polar effects on the expression of the downstream *ess *genes, nor on the divergently transcribed gene directly upstream of *esxA*, predicted to be involved in staphyloxanthin synthesis [37,44,45] (data not shown). Our results suggest that *esxA *is located on a monocistronic transcript and is not co-transcribed with the remaining genes of the *ess *gene cluster.

### *esxA *promoter and terminator sequence analysis

In a microarray of strain Newman, *esxA *transcription was found to be upregulated by the σ^B^-controlled *yabJ-spoVG *operon [10]. Searching the nucleotide sequence upstream of the *esxA *ORF for potential σ^A ^(TTGACA-16/18-TATAAT) [46,47] and σ^B ^(GTTTAA-12/15-GGGTAT) [30] consensus promoter sequences and for a ribosomal binding site (AGGAGG) [48], we identified 80 bp upstream of *esxA *a putative σ^A ^promoter (TatACA-17-TATtAT), and 155 bp upstream of *esxA *a potential σ^B ^promoter (GgTTAA-12-GGGTAT). A proposed ribosomal binding site (RBS, AGGAGG) was located 9 bp upstream of the *esxA *start codon (Figure 1A). Fourteen bp downstream of the *esxA *stop codon we identified a putative Rho independent terminator consisting of a 13 bp inverted repeat with a minimal free energy ΔG of -17 kcal/mol as calculated by mfold [49].

Primer extension analysis located the transcriptional start point (TSP) of *esxA *74 bp upstream of the start codon of *esxA *(Figure 1A-C). It was preceded by the predicted -10 and -35 σ^A ^promoter elements, and further up by the σ^B ^promoter.

**Figure 2:**
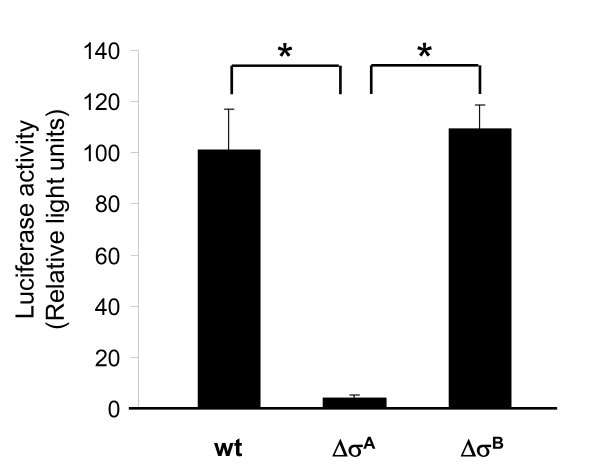
**σ^A^-dependence of the *esxA *promoter**. Luciferase activities of plasmids p*esxAp*-*luc*^+ ^(wt), p*esxAp*Δσ^A^-*luc^+ ^*(Δσ^A^) and p*esxAp*Δσ^B^-*luc*^+ ^(Δσ^B^) in *S. aureus *Newman. The strains were grown in LB broth at 37°C and 180 rpm for 3 h. Data shown are the means ± SD of four independent experiments. Statistical significances between the different strains were assessed with a paired, two-tailed Student's *t*-test (* p < 0.01).

To verify and compare the function of the putative σ^A ^and σ^B ^promoter sequences, we cloned the *esxA *promoter region upstream of the firefly luciferase reporter gene and analyzed the luciferase activity of this construct, p*esxAp-luc^+^*, as well as of constructs containing either a deletion of the σ^A ^or σ^B ^promoter (p*esxAp*Δσ^A^*-luc^+^*, p*esxAp*Δσ^B^*-luc^+^*). Whereas the relative luciferase activities of p*esxAp-luc^+ ^*and p*esxAp*Δσ^B^*-luc^+ ^*after 3 h of growth were comparable, p*esxAp*Δσ^A^*-luc^+ ^*showed almost no activity, suggesting that *esxA *possesses a σ^A^-dependent promoter (Figure 2). We could rule out a direct involvement of σ^B ^in the control of the *esxA *promoter, furthermore, by testing the *esxA *upstream region in the heterologous two-plasmid system that was established to identify σ^B^-dependent *S. aureus *promoters [30]. The upstream region of *esxA *was cloned into the reporter plasmid pSB40N resulting in plasmid p*esxAp *which then was introduced into *E. coli *DH5α containing either pAC7-*sigB*, expressing the *S. aureus sigB *gene from an inducible promoter, or the empty plasmid pAC7. If the *S. aureus *σ^B ^- *E. coli *RNA polymerase core enzyme hybrid recognized the *esxA *promoter, dark blue colonies would be expected on the indicator LBACX-ARA agar [29] in combination with pAC7-*sigB*, as with the σ^B^-dependent promoters of *asp23 *or *yabJ *(positive controls); if not, uncolored colonies would be expected, as with the σ^B^-independent promoter of *capA *or the empty pSB40N (negative controls). In contrast, transformants containing the empty pAC7 vector should produce uncolored colonies. However, both combinations, p*esxAp *with either pAC7 or pAC7-*sigB*, developed an identical only light blue color in *E. coli *DH5α, indicating that the *esxA *promoter was recognized weakly by an *E. coli *RNA polymerase, but that the observed transcriptional activity was independent from σ^B ^(data not shown). Overall, the results of the *esxA *promoter and terminator sequence analyses supported a monocistronic transcription of *esxA *from a σ^A^-dependent promoter.

### Effect of σ^B ^and σ^B^-controlled SpoVG on *esxA *expression

**Figure 3:**
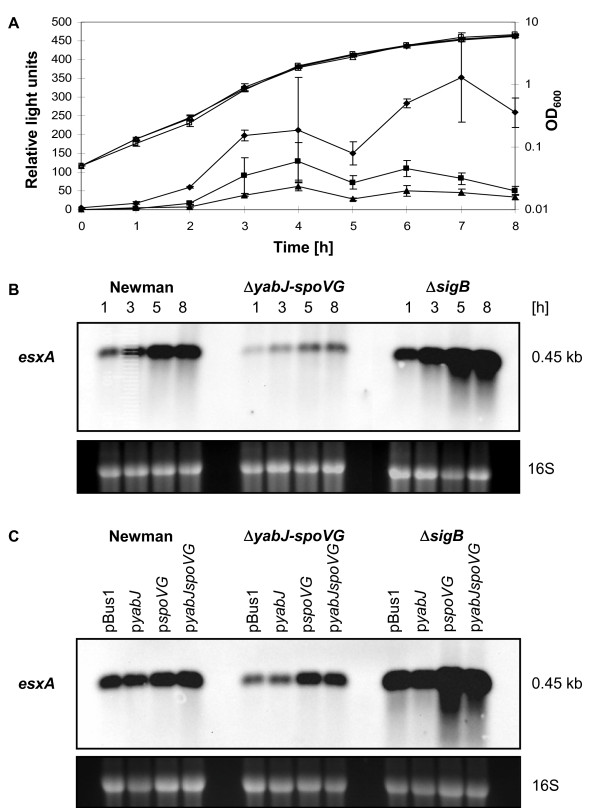
**Effect of σ^B ^and σ^B^-controlled SpoVG on *esxA *expression**. **A**. Transcriptional activity of the *esxA *promoter in strain Newman (squares), SM148 (triangles), and IK184 (diamonds). Growth was followed by measuring the optical density at 600 nm [OD_600_] (open signs), and the activity of the *esxA *promoter was determined by the luciferase activity of p*esxAp-luc^+ ^*(filled signs). **B**. Northern blot analysis of *esxA *transcription in Newman, the Δ*yabJ-spoVG *mutant (SM148) and the Δ*rsbUVW-sigB *mutant (IK184) over growth. **C**. Northern blot showing *esxA *transcription in Newman, the Δ*yabJ-spoVG *mutant (SM148) and the Δ*rsbUVW-sigB *mutant (IK184) complemented with pBus1, p*yabJ*, p*spoVG *or p*yabJspoVG *after 5 h of growth. Ethidium bromide-stained 16S rRNA patterns are shown as an indication of RNA loading.

To differentiate between the effect of σ^B ^and of the σ^B^-controlled *yabJ-spoVG *on the transcriptional control of *esxA*, we followed the luciferase activity of the *esxA *promoter-reporter fusion in p*esxAp-luc^+ ^*during the growth cycle in parental strain Newman, the corresponding Δ*rsbUVW-sigB *mutant (IK184), and in the Δ*yabJ-spoVG *mutant (SM148). The luciferase activity increased in the parent Newman in a growth phase dependent manner from the exponential towards the stationary phase and declined thereafter (Figure 3A). The course of luciferase activity in the Δ*yabJ-spoVG *mutant SM148 and in the Δ*rsbUVW-sigB *mutant IK184 was comparable but the overall activity was reduced by a factor of two in SM148, whereas it was two up to four times higher in IK184. These effects were also mirrored by the intensity of the *esxA *specific transcripts (Figure 3B). Since *esxA *transcription in strain MS64 [24], a mutant with a stop in *sigB *inactivating σ^B^, was indistinguishable from that in IK184, we could assign the upregulation of *esxA *transcription to the loss of σ^B ^and exclude any contributions of *rsbUVW *(data not shown).

To determine if either *yabJ *or *spoVG *inactivation was responsible for the reduction of *esxA *transcription, we complemented Newman, SM148 and IK184 *in trans *with a series of plasmids expressing constitutively either *yabJ *(p*yabJ*), *spoVG *(p*spoVG*), or *yabJ-spoVG *(p*yabJspoVG*), circumventing the requirement of σ^B ^to transcribe the *yabJ-spoVG *operon. Northern blot analysis revealed that the constructs containing *spoVG *or *yabJ-spoVG*, but not the one carrying *yabJ*, did restore the *esxA *transcription to wild type level in SM148 (Figure 3C). In IK184, showing stronger *esxA *transcription signals than the wild type, the *esxA *transcription was even further enhanced by the complementation with p*spoVG *or p*yabJspoVG*, confirming that SpoVG, but not YabJ, had a positive effect on *esxA *expression in presence and absence of σ^B^. However, the fact that *esxA *transcription was strongly induced in IK184 lacking not only σ^B^, but consequently also the σ^B^-dependent SpoVG, suggested that *esxA *transcription may be activated by SpoVG but repressed by other σ^B^-dependent factors.

### Influence of major regulators SarA, RNAIII and ArlR on *esxA*

**Figure 4:**
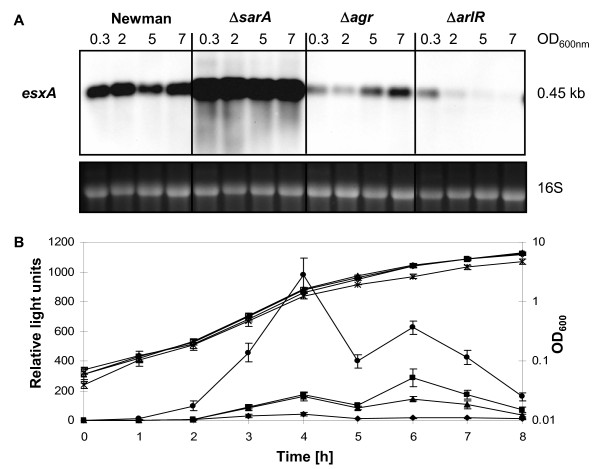
**Effect of SarA, *agr *and ArlR on *esxA *expression**. **A**. Northern blot of *esxA *in Newman, and the Δ*sarA *(LR15), Δ*agr *(KS186) and Δ*arlR *(SM99) mutants over growth. The ethidium bromide-stained 16S rRNA pattern is shown as an indication of RNA loading. **B**. Transcriptional activity of the *esxA *promoter in strain Newman (squares), Δ*sarA *mutant BS309 (stars/dots), Δ*agr *mutant BS310 (triangles), and Δ*arlR *mutant SM99 (diamonds). Growth was followed by measuring the OD_600 _(open signs), and the activity of the *esxA *promoter-reporter construct was determined by the luciferase activity of p*esxAp-luc^+ ^*(filled signs). The strains BS309 and BS310 are isogenic to LR15 and KS186, respectively, except for an exchanged resistance marker in the inactivated loci allowing the selection and maintenance of p*esxAp-luc^+ ^*.

As σ^B ^and SpoVG had opposite effects on *esxA *expression, we searched for further σ^B^-dependent regulators that might be involved in *esxA *control, namely the two major regulators of *S. aureus*, the *agr *system with its effector molecule RNAIII; and the transcriptional regulator SarA. A further candidate was ArlR, the response regulator of the ArlRS two-component system, reported to be activated by σ^B ^in strain Newman, and promoting together with SpoVG capsule formation [9]. The transcript intensity of *esxA *in Newman compared to that in its isogenic Δ*sarA *(LR15), Δ*agr *(KS186) and Δ*arlR *(SM99) mutants during growth, revealed a strong upregulation of *esxA *in LR15, a downregulation in KS186 and an even stronger attenuation in SM99 (Figure 4A), suggesting that SarA acts as repressor, and RNAIII and ArlR as activators of *esxA *transcription. This was confirmed by the level of luciferase activity of p*esxAp-luc^+ ^*during growth, which was highly increased in the Δ*sarA *mutant (BS309), and lower in the Δ*agr *(BS310) and almost absent in Δ*arlR *(SM99) mutants compared to the wild type Newman (Figure 4B). Interestingly, as in capsule synthesis, SpoVG and ArlR acted as elements enhancing the *esxA *expression [9].

### Influence of EsxA on regulatory elements and itself

EsxA itself had no influence on the signal intensity or activity of any of the above regulatory genes, neither on *asp23*, as an indicator of σ^B ^activity [37,44,50], nor on *spoVG, arlR*, *sarA *or RNAIII, when comparing their expression in strain Newman and in the Δ*esxA *mutant BS304 during the growth cycle (Additional file 1). We could also rule out any autoregulatory effects of EsxA on its own transcription, since luciferase activity patterns of p*esxAp-luc^+ ^*were congruent over the entire growth cycle in Newman and BS304 (data not shown).

### Influence of SarA, RNAIII, σ^B^, ArlR and SpoVG on each other

**Figure 5:**
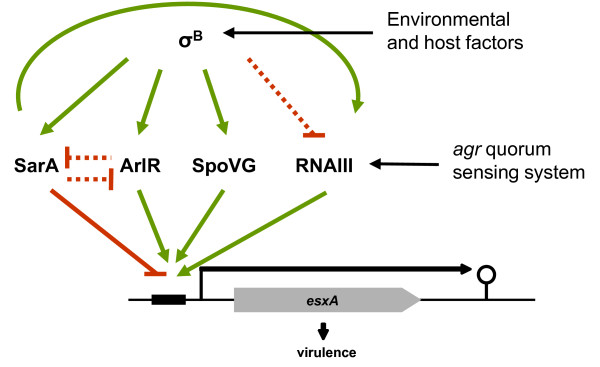
**Transcriptional regulation of *esxA *by global regulators of virulence in strain Newman**. Major upregulation is represented by green arrows, downregulation by red bars. Dashed lines indicate minor influences.

An overview of the regulatory network influencing *esxA *transcription is given in Figure 5, including also the mutual interactions of the different regulators: σ^B ^activity was found to be comparable in all strains tested, excluding secondary effects on *esxA *transcription due to an altered σ^B ^activity (Additional file 2). We confirmed the previously reported positive influence of σ^B ^on *arlRS *and *yabJspoVG *transcription [7,9], as well as on *sarA *transcription [3,7]. In contrast, we could not detect any major changes in RNAIII transcript intensity in σ^B ^mutants, although some studies suggest that σ^B ^activity is reducing the RNAIII level [3,4] (Additional file 2).

Further, minor changes in transcription were observed in the Δ*sarA *mutant where RNAIII was downregulated and *arlR *transcripts were slightly upregulated, and in the Δ*arlR *mutant where *sarA *transcription was increased (Additional file 2: Figure S2A). However, these dependencies could not explain the changes in *esxA *transcription in the corresponding mutants.

### Phenotypic characteristics of the Δ*esxA *mutant

The successful deletion of *esxA *reported here, and the superimposable growth rates of wild type and *esxA *mutant in complex LB medium, confirmed that EsxA was not essential for growth in vitro (data not shown). The growth defects observed in *sigB *and *arlR *mutants, the former affecting late [37] and the latter reducing early growth stages [19], can therefore also not depend on altered EsxA expression. Although σ^B ^and SpoVG are known to influence extracellular proteolytic activities [9], and σ^B ^is known to repress hemolytic activity in *S. aureus *[4,7,37], EsxA did neither affect proteolytic nor hemolytic activities in BS304 (data not shown). As the activity of the sigma factor σ^B ^and the σ^B^-controlled SpoVG positively influences methicillin and glycopeptide resistance in methicillin resistant *S. aureus *(MRSA) and in glycopeptide intermediate resistant *S. aureus *(GISA) [8,51-55], we deleted *esxA *in MRSA strain BB1002 [26] and GISA strain NM143 [27]. However, resistance levels of the Δ*esxA *mutants BS307 and BS308 to oxacillin and teicoplanin, respectively, were identical to those of the parent strains, when measured by Etest (Table 3), as well as by antibiotic gradient plates, which allow the detection of very small differences in resistance (data not shown). These results suggest that EsxA, which enhances abscess formation in mice and is thought to act either as transport chaperone or adaptor protein [18], primarily plays a role as extracellular virulence factor in pathogenesis.

**Table 3:** Oxacillin and teicoplanin MICs

Strain	MIC (μg ml^-1^)	
	
	Oxacillin	Teicoplanin
Newman	0.19	4

BS304	0.19	4

BB1002	> 256	3

BS307	> 256	3

NM143	0.25	12

BS308	0.25	12

## Conclusion

Our data suggest that the repression of *esxA *by σ^B ^is due the σ^B^-induced transcription of *sarA*, leading to a strong and dominating SarA-mediated repression of *esxA*. The activation of *esxA *transcription, on the other hand, is stimulated by the *agr *quorum sensing system, the response regulator ArlR, and the effector protein SpoVG; whereby *arlR *is controlled indirectly, and *spoVG *directly by σ^B^. Thereby the activating effect of ArlR seems to be more profound than the effect of SpoVG and *agr*. Moreover, virulence gene regulation in *S. aureus *is very complex and additional factors might contribute to the regulation of *esxA *transcription.

The mode of function of SpoVG, named after the stage V sporulation protein G in *Bacillus subtilis *[7], and SpoVG homologues in other bacterial species is yet unknown, nor have any SpoVG interacting partners been reported. SpoVG does not affect σ^B ^activity as seen from the expression of *asp23*, which is a measure of σ^B ^activity in *S. aureus*. SpoVG does also not interfere with the transcription of *sarA*, *arlRS *nor *agr *in strain Newman.

By which mechanisms SpoVG counteracts the postulated SarA-mediated repression of *esxA *remains open. The affinity of SarA binding to DNA can be enhanced by phosphorylation [56], but a postulated interaction of SpoVG with SarA or other proteins has yet to be investigated. Interestingly, the same stimulating effect by ArlRS and SpoVG is seen in *S. aureus *capsule synthesis [9]. We therefore can not rule out that SpoVG and ArlR may interact or have some common target. SpoVG by itself seems also to enhance transcription of *esxA *when artificially overexpressed in a *sigB *mutant. The absence of predicted DNA binding motifs in SpoVG may not fully exclude its interaction with nucleic acids or with factors involved in transcription. In conclusion, we have presented here SpoVG, an interesting new player in the regulatory cascade modulating *S. aureus *virulence factors.

## Authors' contributions

BS carried out most of the experiments, participated in the design of the study and drafted the manuscript. DAB participated in the transcriptional analysis. BBB conceived the study, and participated in its design and coordination, and helped to draft the manuscript. All authors read and approved the final manuscript.

## Supplementary Material

Additional file 1 **No influence of EsxA on *asp23, arlR, sarA, spoVG *and RNAIII transcription**. Northern blot analysis comparing the transcript intensities of *asp23, arlR, sarA, spoVG *and RNAIII in *S. aureus *Newman and its Δ*esxA *mutant. Click here for file

Additional file 2 **Influence of SarA, RNAIII, σ^B^, ArlR and SpoVG on each other**. Northern blot analysis comparing the transcript intensities of *asp23, arlR, sarA, spoVG *and RNAIII in *S. aureus *Newman, and its isogenic Δ*sarA*, Δ*agr*, Δ*arlR*, Δ*yabJspoVG *and Δ*rsbUVW-sigB *mutant, respectively. Click here for file
