# Supplementary material for: Opposing roles of σB and σB-controlled SpoVG in the global regulation of esxA in Staphylococcus aureus
Source: BMC Microbiol. 2012 Jan 24;12:17. doi: 10.1186/1471-2180-12-17 (PMC3313859; doi:10.1186/1471-2180-12-17)
Supplement: Additional file 1 — No influence of EsxA on asp23, arlR, sarA, spoVG and RNAIII transcription. Northern blot analysis comparing the transcript intensities of asp23, arlR, sarA, spoVG and RNAIII in S. aureus Newman and its ΔesxA mutant. [file 1471-2180-12-17-S1.PDF]

ADDITIONAL FILE 1

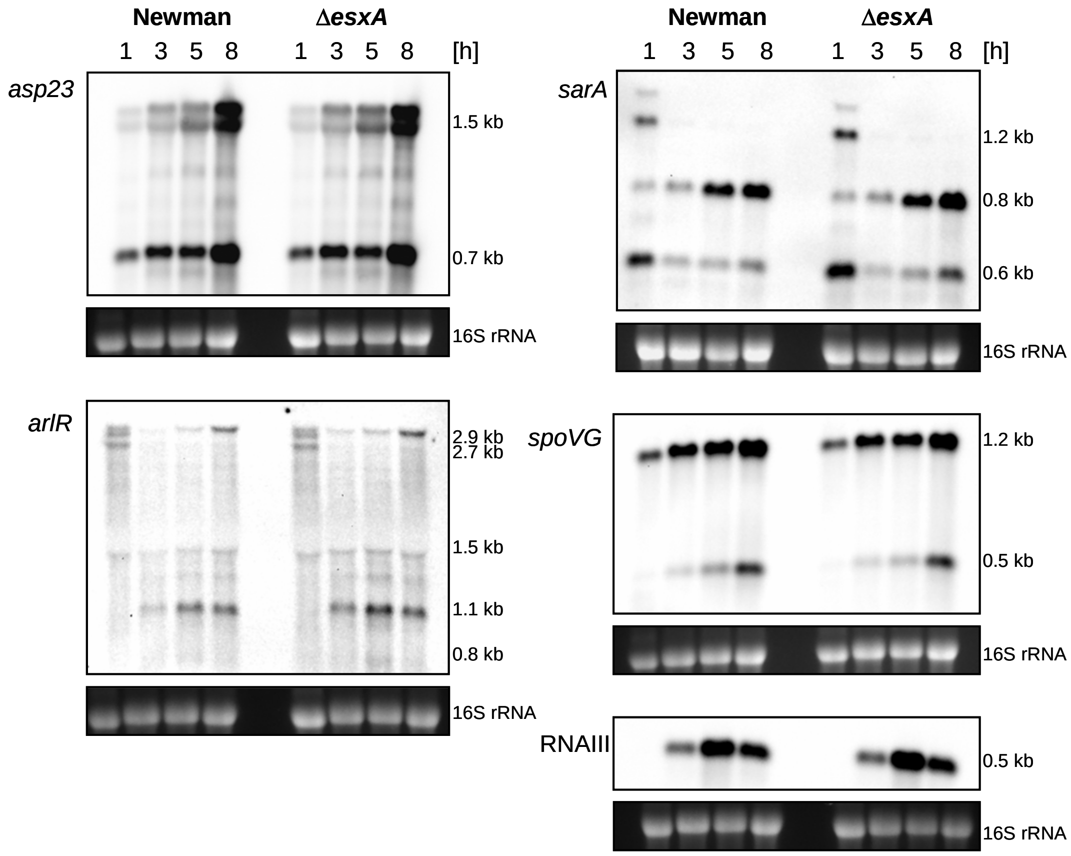

Additional figure 1. No influence of EsxA on *asp23, arlR, sarA, spoVG* and RNAIII transcription. Northern blot analysis comparing the transcript intensities of *asp23, arlR, sarA, spoVG* and RNAIII in *S. aureus* Newman and its Δ*esxA* mutant BS304 grown for 1, 3, 5, and 8 h in LB broth at 37°C. Transcript sizes are indicated. The ethidium bromide stained 16S rRNA pattern is shown as an indication of the RNA loading.
