# Supplementary material for: Opposing roles of σB and σB-controlled SpoVG in the global regulation of esxA in Staphylococcus aureus
Source: BMC Microbiol. 2012 Jan 24;12:17. doi: 10.1186/1471-2180-12-17 (PMC3313859; doi:10.1186/1471-2180-12-17)
Supplement: Additional file 2 — Influence of SarA, RNAIII, σB, ArlR and SpoVG on each other. Northern blot analysis comparing the transcript intensities of asp23, arlR, sarA, spoVG and RNAIII in S. aureus Newman, and its isogenic ΔsarA, Δagr, ΔarlR, ΔyabJspoVG and ΔrsbUVW-sigB mutant, respectively. [file 1471-2180-12-17-S2.PDF]

ADDITIONAL FILE 2

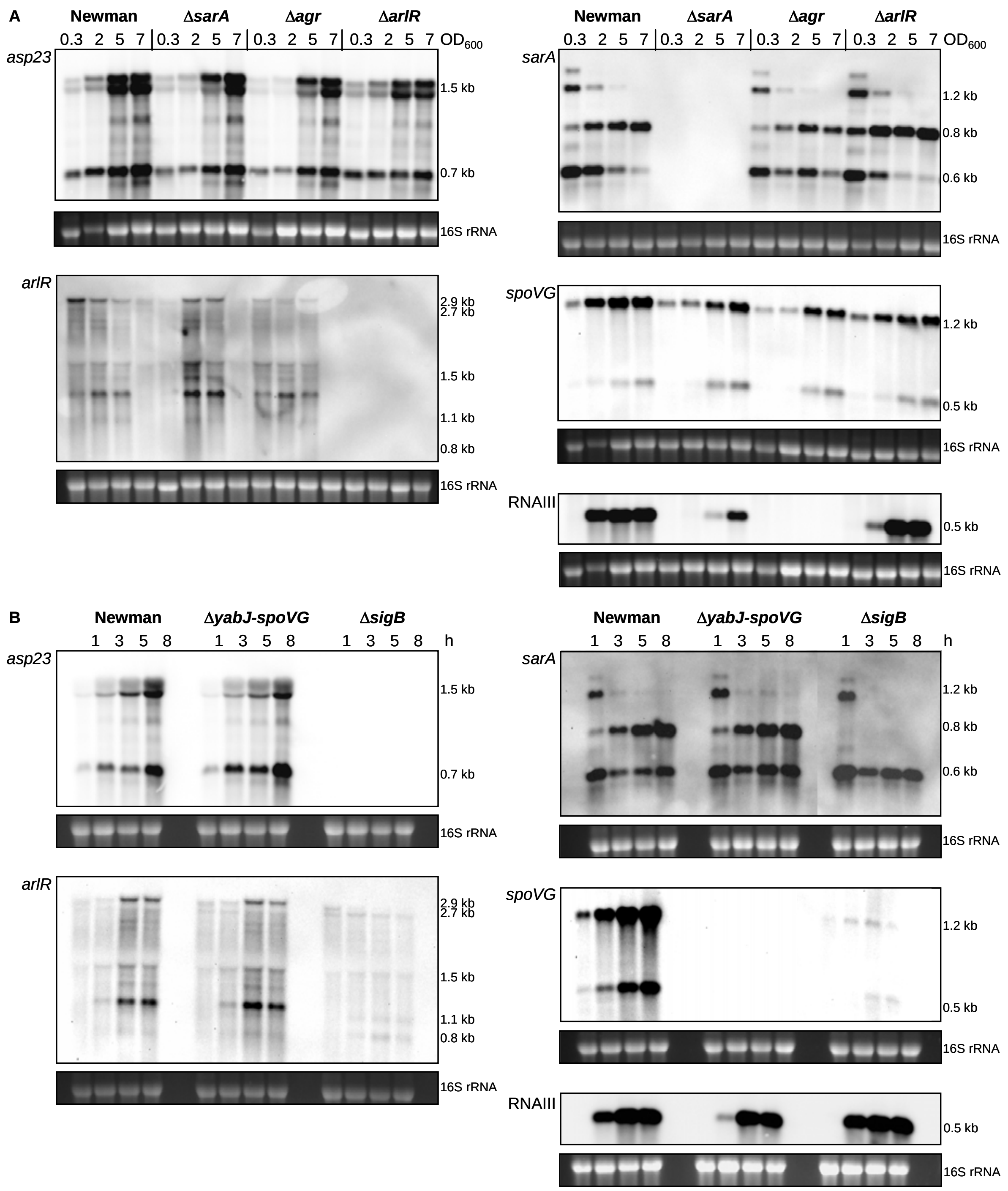

Additional figure 2. Influence of SarA, RNAIII, σ^B, ArlR and SpoVG on each other. Northern blot analysis comparing the transcript intensities of *asp23, arlR, sarA, spoVG* and RNAIII in *S. aureus* Newman, its Δ*sarA* mutant LR15, its Δ*agr* mutant KS186 and its Δ*arlR* mutant SM99 (A), and in *S. aureus* Newman, SM148 (Δ*yabJspoVG*) and IK184 (Δ*rsbUVW-sigB*) (B). Transcript sizes are indicated. The ethidium bromide stained 16S rRNA pattern is shown as an indication of the RNA loading.
